# Chronic Occupational Exposure to Chemical Mixtures Induces Genomic Instability in Paint Workers

**DOI:** 10.3390/toxics13090785

**Published:** 2025-09-17

**Authors:** Servet Birgin İritaş, Merve Güdül Bacanlı, Gökçe Taner, Vugar Ali Türksoy, Lütfiye Tutkun, Ömer Hınç Yilmaz, Ayşe Nurşen Başaran

**Affiliations:** 1 Department of Forensic Medicine, Faculty of Medicine, Ankara Yıldırım Beyazıt University, 06800 Ankara, Türkiye; 2 Department of Pharmaceutical Toxicology, Faculty of Pharmacy, Health Sciences University, 06018 Ankara, Türkiye; merve.gudulbacanli@sbu.edu.tr; 3 Department of Bioengineering, Faculty of Engineering and Natural Sciences, Bursa Technical University, 16310 Bursa, Türkiye; gokce.taner@btu.edu.tr; 4 Department of Public Health, Faculty of Medicine, Yozgat Bozok University, 66100 Yozgat, Türkiye; v.aliturksoy@yobu.edu.tr; 5 The Association of Industrial Toxicology and Occupational Hygiene, 06660 Ankara, Türkiye; lutfiye.tutkun@etok-ihider.org; 6 Department of Public Health, Faculty of Medicine, Ankara Yıldırım Beyazıt University, 06800 Ankara, Türkiye; ohyilmaz@aybu.edu.tr; 7 Department of Pharmaceutical Toxicology, Faculty of Pharmacy, Başkent University, 06800 Ankara, Türkiye; anbasaran@baskent.edu.tr

**Keywords:** paint workers, occupational exposure, DNA damage, comet assay, buccal micronucleus assay, trichloroacetic acid, hippuric acid, phenol, lead

## Abstract

This study’s objective was to evaluate genotoxic effects on automotive paint workers who are exposed to a complex mixture of VOCs, heavy metals, and solvents. Biological samples, including blood, urine, and buccal epithelial cells, were collected from 80 exposed workers and 80 demographically matched control subjects. DNA damage was assessed using the alkaline COMET assay in lymphocytes and whole blood. The Buccal Micronucleus Cytome (BMCyt) assay was also employed to identify cytogenetic abnormalities. Additionally, trichloroacetic acid (TCA), hippuric acid (HA), phenol, and lead (Pb) levels were measured as biomarkers of exposure. A significant increase in DNA damage was observed in the lymphocytes and whole blood of exposed workers (*p* < 0.05) BMCyt analysis also revealed higher frequencies of micronuclei (MN), binucleated cells, condensed chromatin (CC), and karyorrhectic (KHC) and pyknotic cells (PYC) in buccal cells (*p* < 0.05). Elevated levels of urinary HA, phenol, TCA, and blood lead indicated systemic chemical exposure. DNA damage positively correlated with these biomarkers, supporting a strong link between chronic occupational exposure and genotoxicity. The findings from this study highlight the critical importance of implementing effective safety measures and consistent biomonitoring for paint workers to prevent adverse health effects.

## 1. Introduction

Paint industry workers are often exposed to a wide variety and quantity of chemicals, including heavy metals (e.g., lead, cadmium, zinc, chromium, and cobalt); aliphatic alcohols (such as methanol, isopropanol, and butanol); aromatic hydrocarbons (such as benzene, toluene, xylene, and trimethylbenzene); aliphatic hydrocarbons (such as hexane and heptane); and silica crystals (such as quartz) [1]. Toluene is among the most frequently utilized industrial chemicals. Upon occupational exposure, the substance undergoes primary metabolism in the liver, where it is converted to benzoic acid. This acid is subsequently conjugated with glycine, which results in the formation of HA. This metabolite is then excreted in urine. Studies have demonstrated that approximately 75–80% of the absorbed toluene is eliminated as HA in urine within 24 h, making it a useful biomarker for recent toluene exposure [2,3]. Recent investigations conducted in various industrial settings, such as in automotive refinishing and thinner production, have confirmed a strong correlation between ambient toluene concentrations and urinary HA levels. This relationship supports the use of HA as a reliable biomarker for the biological monitoring of toluene exposure [3,4]. Inhalation of toluene, benzene, xylene, and similar organic solvents leads to the generation of reactive oxygen species (ROS). The resulting oxidative stress and lipid peroxidation in cellular membranes play a significant role in the initiation and progression of various diseases [5,6]. Such exposures have been reported to result in serious cardiac arrhythmia, chronic toxic encephalopathy, and seizures, ultimately contributing to increased mortality and morbidity rates [7,8,9].

Increased formation of ROS has been reported to decrease antioxidant enzyme levels, increase DNA damage, and/or impair DNA repair mechanisms. Oxidative stress can lead to single- and double-strand breaks, basic sites, base modifications, and sugar damage in DNA [10], and if this damage is not repaired, it can lead to aging, mutagenesis, carcinogenesis, and cell death [11].

Recent studies have shown that painters and other occupational groups are at high risk of exposure to toluene and other VOCs, leading to significant increases in urinary biomarkers. For example, in a study conducted on workers in a paint manufacturing facility, HA concentrations were found to range between 217.2 and 4045.5 mg/g creatinine. Although no biological exposure indices (BEIs) for urinary HA have been established following the reduction of the ACGIH-TLV to 20 ppm, the detected levels were reported to be higher than the former ACGIH BEI (<1.6 g/g creatinine) (ACGIH, 2007) [3,12,13,14,15].

Biomonitoring can be used to detect occupational exposure to harmful compounds such as volatile organic solvents and heavy metals in the paint industry, by analyzing specific metabolites or trace elements in biological fluids. TCA is a well-known urinary biomarker for exposure to chlorinated solvents such as trichloroethylene, which are commonly found in degreasers and paint strippers. The accumulation of TCA in urine reflects oxidative metabolism and indicates systemic exposure to trichloroethylene and related compounds [16].

Hippuric acid is a widely accepted metabolite of toluene, a major component of many solvent-based paints. Despite some controversies regarding its specificity, it remains a cost-effective biomarker for biological monitoring in occupational settings, especially when coupled with air concentration measurements [3,4]. Phenol, derived from the metabolic biotransformation of benzene, serves as a non-specific biomarker that indicates exposure to aromatic hydrocarbons. Increased urinary phenol levels are associated with DNA damage and oxidative stress, supporting its use in assessing genotoxicity risk [17]. Blood lead levels are a well-established and reliable biomarker of chronic exposure to inorganic lead, which may still be present in paints and primers despite regulatory restrictions. Elevated blood lead levels have been linked to increased DNA damage, immunotoxicity and neurobehavioral disorders, highlighting the importance of regular biomonitoring in high-risk occupational groups [14,18].

These findings underscore the necessity of monitoring workers in the paint industry for multiple chemical exposures using a combination of biomonitoring and hematological/oxidative stress markers. Biomonitoring provides a valuable tool to assess occupational exposure to hazardous substances such as VOCs and heavy metals through the analysis of specific metabolites or trace elements in biological fluids.

This study’s objective was to evaluate the genotoxic effects on automotive paint workers who are exposed to a complex mixture of VOCs, heavy metals, and solvents. To accomplish this, a dual biomonitoring strategy was employed; DNA strand breaks were assessed in both whole blood and peripheral blood lymphocytes using the alkaline COMET assay. The Buccal Micronucleus Cytome (BMCyt) assay was employed to detect cytogenetic abnormalities such as MN and nuclear anomalies in exfoliated buccal epithelial cells. In addition to genotoxicity assays, levels of blood lead, urinary TCA, urinary HA (for toluene exposure), and urinary phenol (for aromatic hydrocarbon exposure) were quantified. All biomarker and cytogenetic data from occupationally exposed auto-motive paint workers were systematically compared with a demographically matched control group to determine the extent of genotoxic alterations attributable to occupational chemical exposure.

## 2. Materials and Methods

### 2.1. Subjects

A total of 80 male volunteer workers from the car painting industry in Ankara, Turkey, who had worked in the field for at least one year, as the case group, and 80 male volunteer office workers with no history of occupational chemical exposure, as the control group, were included in this study. Before sample collection, all volunteers were informed, their consent was obtained, and they completed a comprehensive questionnaire to record their personal data. Those with acute or chronic illnesses or taking medical or dietary medications were excluded from this study. To minimize the influence of lifestyle variables, all participants were selected from the same region.

### 2.2. Chemicals and Equipment

The experimental chemicals and materials were obtained from different suppliers. Normal melting agarose (NMA) and low melting agarose (LMA) were purchased from Boehringer Mannheim, Germany. Reagents including sodium chloride (NaCl), sodium hydroxide (NaOH), activated charcoal, methanol, ethanol, hydrochloric acid (HCl), sodium bisulfite, pararosaniline, fast green, xylene, and ultrapure nitric acid were obtained from Merck Chemicals in Darmstadt, Germany. Sigma (Missouri, USA) supplied hydrogen peroxide (H_2_O_2_), dimethyl sulfoxide (DMSO), ethidium bromide (EtBr), Triton X-100, and PBS tablets. Additionally, ethylenediaminetetraacetic acid disodium salt dihydrate (Na_2_EDTA), sodium lauroyl sarcosinate, and Tris(hydroxymethyl)aminomethane were purchased from ICN Biomedicals, Ohio, USA. The lead standard used for ICP-MS analysis (TraceCERT^®^, 1 mg/L Pb in nitric acid) was obtained from Sigma-Aldrich, Darmstadt, Germany.

### 2.3. Single Cell Gel Electrophoresis (Comet) Assay

Approximately 3 mL of whole blood was collected from the antecubital vein using a sterile stainless-steel needle and placed in heparinized tubes. This was then mixed with an equal volume of ice-cold PBS, after which the lymphocytes were separated using the Ficoll Hypaque density gradient centrifugation method [19]. A total of 500 µL of whole blood was used directly for the comet assay. Cell viability was determined by trypan blue exclusion, after which the cell concentration was adjusted to ~2 × 10^5^ cells/mL in buffer. All procedures were performed on the same day as blood collection.

The comet assay was conducted according to the alkaline protocol originally de-scribed by Singh et al. [20] and subsequently modified by Anlar et al. [21]. The lymphocytes and whole blood cells were embedded in agarose on slides, lysed, and then subjected to electrophoresis to separate the fragmented DNA, which gave them a ‘comet’-like appearance. After electrophoresis, the slides were neutralized and then subjected to dehydration through a graded ethanol series (50%, 75%, and 98%), with each step lasting five minutes. Once air-dried, the slides were stained with ethidium bromide (20 µg/mL; 60 µL per slide) and examined under green light using a Leica^®^ fluorescence microscope. Images were captured with a CCD camera connected to a computer system and analyzed using Comet Analysis Software (version 3.0, Kinetic Imaging Ltd., Liverpool, UK). DNA damage was quantified by measuring the percentage of DNA present in the comet tail (tail intensity), evaluating 100 nuclei per sample at 40× magnification.

### 2.4. Buccal Micronucleus Cytome Assay (BMCyt Assay)

Participants were instructed to rinse their mouths with water before providing a sample. The inner lining of both cheeks was gently scraped with a wet tongue depressor to obtain buccal epithelial cells. The cells were then smeared immediately onto premoistened microscope slides and left to air-dry. On the same day, the slides were fixed in 80% methanol and processed further in the laboratory. Staining was performed according to the protocol described by Kashyap & Reddy [22]. The slides were treated with 1 N HCl for two minutes at room temperature, incubated at 60 °C for ten minutes, and rinsed again at room temperature. After washing with distilled water and drying, Feulgen staining solution (1 g pararosaniline dissolved in 100 mL distilled water) was applied for 90 min in the dark. This was followed by a brief rinse and counterstaining with fast green (0.5 g in 95% ethanol) for 10 s. The slides were then immersed in xylene for 10 min and air-dried. The BMCyt assay was then employed to identify biomarkers of genomic instability, including MN, nuclear buds (NBUD), and indicators of cell death, such as pyknotic (PYC), karyorrhectic (KHC), condensed chromatin (CC), and karyolytic (KYL) cells, as well as binucleated cells (BN). As outlined by Thomas et al. [23], 2000 cells per individual (1000 cells from two slides) were analyzed using light microscopy. The results were expressed as frequencies per thousand cells.

### 2.5. Biomarker Analysis for Occupational Exposure

To evaluate internal exposure to toxic compounds among paint workers, biological monitoring was performed by measuring selected urinary and blood biomarkers, specifically TCA, HA, phenol in urine, and blood lead.

Morning spot urine samples were collected in sterile polypropylene containers and stored at −20 °C until analysis. Urinary TCA concentrations were measured using head-space gas chromatography equipped with a flame ionization detector (HS-GC-FID), as described by Ogata et al. [24,25]. Hippuric acid and phenol levels were simultaneously determined using high-performance liquid chromatography (HPLC) with UV detection at 254 nm, following derivatization with dansyl chloride. Quantification was based on calibration curves prepared with analytical-grade standards, and creatinine-adjusted values were used to normalize inter-individual variations in urinary dilution.

Five milliliters of venous blood were drawn into EDTA tubes free of metal contamination and kept at 4 °C until analysis. Lead levels in the blood were determined using inductively coupled plasma mass spectrometry (ICP-MS) after wet acid digestion. For each assay, 1 mL of blood was subjected to closed-vessel microwave-assisted acid digestion within Teflon digestion tubes, employing a reagent mixture composed of 5 mL ultrapure nitric acid (HNO_3_) and 5 mL ultrapure deionized water. Post-digestion, the resulting solution was quantitatively diluted to a final volume of 20 mL with ultrapure water to ensure matrix compatibility for ICP-MS analysis. All procedural steps, including the preparation of calibration standards and sample dilutions, were conducted exclusively with ultrapure water to eliminate background contamination. Pb analysis was performed on a Thermo Scientific iCAP Qc ICP-MS system (USA), operated under optimized instrumental parameters to maximize detection efficiency and minimize matrix interference. Prior to analysis, the system was thoroughly cleaned using a sequence of ultrapure water and trace metal grade nitric acid, ensuring baseline stabilization. Lead quantification employed a 7-point external calibration curve spanning concentrations from 0.1 to 100 µg/L, yielding excellent linearity (correlation coefficient r = 0.9999). The method demonstrated a limit of detection (LOD) of 0.0038 µg/L and a procedural blank (background level) of 0.011 µg/L. To ensure analytical robustness, all sample and standard measurements were performed in triplicate, with a resulting relative standard deviation (RSD) consistently below 5%. Certified reference materials (CRMs) were employed for quality control, and all analyses were performed in accordance with the guidelines of the U.S. Centers for Disease Control and Prevention [26].

### 2.6. Statistical Analysis

Statistical analyses were performed with SPSS software, version 23.0 (IBM, New York, USA). Data normality was examined using the Kolmogorov–Smirnov test, and homogeneity of variance was evaluated with Levene’s test. Continuous data are expressed as mean ± standard deviation (SD), while categorical data are shown as percentages. For categorical comparison, we used the Pearson Chi Square test. Continuous data were compared between two groups using Student’s *t*-test. A *p*-value of less than 0.05 was considered statistically significant.

## 3. Results

### 3.1. Characteristics of the Study Groups

A total of 80 exposed workers and 80 control individuals, matched based on demographic variables, provided blood, urine, and buccal cell samples. The participants’ demographic information is presented in Table 1. There were no statistically significant differences in age distribution between the exposed group and the controls (mean age: 39.00 ± 7.17 years vs. 37.83 ± 8.16 years; *p* > 0.05). However, smoking rates were notably higher in the exposed group (56.25%) compared to the control group (35%), and this difference reached statistical significance. Notably, none of the participants in either group reported consuming alcohol.

### 3.2. Comet Assay

The extent of DNA damage, as determined by the COMET assay, was significantly higher in the exposed group than in the control group. As shown in Table 2, the mean tail intensity in lymphocytes was significantly higher among workers at 5.00 ± 0.50% than in controls at 2.69 ± 1.05% (*p* < 0.05). Similarly, elevated whole blood tail intensity values were observed in workers (7.65 ± 3.47%) compared to controls (5.48 ± 2.41%; *p* < 0.05), indicating a systemic genotoxic insult. Subgroup analysis revealed that DNA damage was consistently higher across all age groups and smoking statuses within the exposed group. However, no statistically significant relationship was identified between DNA tail intensity and participants’ age, smoking status, or exposure duration (*p* > 0.05). Despite workers’ claims regarding the use of personal protective equipment (PPE), qualitative data from interviews suggested that such equipment was used insufficiently or was ineffective.

### 3.3. Buccal Micronucleus Cytome Assay Results (BMCyt Assay)

As presented in Table 3, buccal cell micronucleus frequency, as determined by the BMCyt assay, was significantly higher in the worker group than in the control group (3.23 ± 2.71 vs. 2.36 ± 1.93; *p* < 0.05). While no significant associations were found with age or smoking status, workers with longer exposure durations (18–35 years) exhibited notably higher MN frequencies (4.38 ± 4.63‰) than those with 1–17 years of exposure (2.93 ± 1.41‰). This suggests a potential cumulative genotoxic effect (*p* < 0.05).

**Figure 1:**
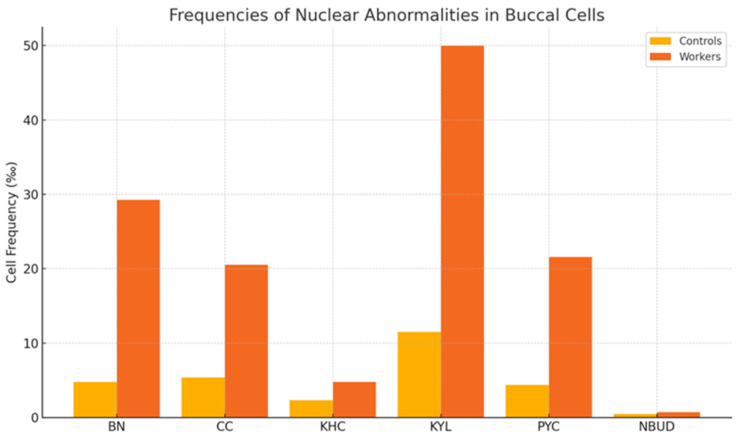
Frequency of different cell types scored in buccal micronucleus cytome asay (BMCyt) ^a^. ^a^ Expressed as BN: binucleated; CC: condensed chromatin; KHC: karyorrhectic; KYL: karyolytic; PYC: pyknotic; NBUD: nuclear bud.

Further examination of cytogenetic endpoints revealed that the frequency of all nuclear abnormalities was significantly higher in individuals exposed to the substance than in the control group (Table 4). Specifically, the frequencies of binucleated cells (BN), condensed chromatin (CC), and karyolytic cells (KYL) increased from 4.80 ± 3.25‰, 5.40 ± 3.63‰, and 11.50 ± 11.10‰ to 29.23 ± 10.32‰, 20.55 ± 18.18‰, and 49.99 ± 5.99‰, respectively (*p* < 0.01). The frequencies of karyorrhectic (KHC), pyknotic (PYC), and nuclear bud (NBUD) cells were also significantly increased, indicating enhanced chromosomal instability and apoptotic activity in buccal epithelial cells (Figure 1 and Table 4).

## 4. Discussion

Findings from the current research indicate a significant increase in DNA damage and cytogenetic abnormalities among paint workers, observed in both buccal epithelial cells and peripheral blood lymphocytes, relative to unexposed controls. These findings are consistent with previous reports in the occupational toxicology literature, highlighting the genotoxic potential of chronic exposure to paint-related chemical mixtures [27,28,29]. Collectively, these biomarkers provide valuable insights into internal exposure levels, thereby supporting the interpretation of cytogenetic assays such as the COMET and MN tests in biomonitoring studies. The significant increases in tail intensity observed in the COMET assay and in MN frequencies in buccal cells of paint workers suggest chronic exposure to genotoxic agents frequently present in paint formulations, including aromatic hydrocarbons, heavy metals, and VOCs [30,31,32]. The elevated DNA damage observed in peripheral blood lymphocytes, as measured by the COMET assay, correlates with increased levels of TCA, HA, phenol, and blood Pb, which serve as biomarkers of exposure to volatile organic compounds and heavy metals [2,3]. Workers exhibited TCA concentrations more than four times higher than those in the control group, indicating a significant metabolic burden resulting from exposure to chlorinated solvents. This exposure has been associated with the generation of free radicals and subsequent oxidative DNA damage [11,33]. Similarly, elevated HA levels, which are indicative of toluene metabolism, correlate with increased tail intensity in both lymphocyte and whole blood DNA. This aligns with studies in which toluene exposure has been shown to cause oxidative stress and genotoxicity in occupational cohorts [5,17,34]. Some studies have also assessed Pb exposure as a significant contributor to genotoxicity. The significantly elevated blood lead levels observed in workers are concerning, given lead’s documented ability to interfere with DNA repair enzymes and promote chromosomal aberrations [35,36]. In our study, lead levels in workers were significantly higher than in the control group but well below risky levels. However, in light of existing studies, it was considered that this difference may contribute to DNA damage. Consequently, the increased prevalence of nuclear abnormalities, such as karyorrhexis and karyolysis, in buccal cells, reinforces the cytotoxic and apoptotic impact of these exposures [37,38]. Furthermore, the BMCyt assay revealed elevated frequencies of binucleated (BN) and pyknotic (PYC) cells, which are markers of cytokinesis defects and cellular degeneration, respectively [37,39]. These findings mirror the elevated levels of phenol, an aromatic metabolite of benzene associated with clastogenic effects and mitotic spindle disruption, observed among workers [13,38]. An important observation is the effect of exposure duration; workers with over 18 years of exposure exhibited significantly higher buccal MN frequencies than those with shorter exposure histories, suggesting a cumulative genotoxic effect [38]. Although the use of protective equipment was reportedly high, field interviews indicated deficiencies in the quality of PPE or its usage, which may have diminished its protective capacity [40]. Overall, this study supports the hypothesis that routine occupational exposure to paint chemicals results in measurable genotoxic outcomes, and that concentrations of biomarkers in biological matrices can serve as proxies for DNA damage risk [23,41,42]. This study aimed to evaluate the genotoxic effects of occupational chemical exposure on workers in the paint industry. COMET analysis revealed increased DNA damage in the workers’ lymphocytes and whole blood, as well as elevated frequencies of MN and other nuclear anomalies in the oral mucosa. These findings suggest that paint workers may sustain significant genetic damage through chronic exposure to complex chemical mixtures. Similarly to prior studies, these findings suggest that contact with volatile organic compounds (e.g., benzene, toluene, xylene) and heavy metals (e.g., cadmium, lead, chromium), which are widely present in paint materials, contributes to DNA damage mediated by oxidative stress [33,40,43]. It is known that compounds found in the paint industry trigger lipid peroxidation by producing ROS in cells, which can lead to DNA damage such as single- and double-strand breaks, base modifications, and protein–DNA cross-links [10]. MN analysis of epithelial cells obtained from the oral mucosa also supported these findings. Significantly higher MN, BN, CC, KHC, KYL, PYC, and NBUD frequencies were observed in workers than in the control group, indicating clastogenic and aneugenic effects [44].

While several studies have identified toluene as a primary contributor to genotoxicity in the paint industry, exposure to complex mixtures of hazardous chemicals—including VOCs, heavy metals, and aromatic hydrocarbons—may further exacerbate oxidative stress-induced cellular damage, ultimately contributing to the development of occupational diseases [45,46]. Such oxidative damage has been associated with an elevated risk of chronic obstructive pulmonary disease (COPD), lung and bladder cancers, and hematological malignancies [34]. Also, several biomonitoring studies have supported these associations. Chang et al. [15] reported oxidative DNA damage and increased urinary 8-hydroxy-2-deoxyguanosine (8-OHdG) levels in spray painters. de Oliveira et al. [31] detected DNA damage in both buccal and blood cells of Brazilian painters using the COMET assay. Londoño-Velasco et al. [32] demonstrated increased oxidative DNA damage in the lymphocytes of car painters via the Fpg-modified COMET assay. These findings are consistent with the increased genotoxicity observed in the current study using both COMET and MN assays. Our results align with previously reported increases in chromosomal aberrations (CAs), sister chromatid exchanges (SCEs), and MN frequencies in the leukocytes, lymphocytes, and buccal epithelial cells of exposed workers [47,48]. Earlier investigations also support this trend [49,50,51].

While most studies confirm the genotoxic effects of occupational paint exposure, findings regarding MN frequency remain inconsistent. Some studies report a significant increase in MN frequencies [25,38,48,50,51,52,53,54], whereas others report no statistically significant difference [32,33]. Cassini et al. [24] observed increased DNA damage via the COMET assay but found no change in MN frequency. However, they did report elevated nuclear budding in lymphocytes and increased condensed chromatin in buccal cells. Moro et al. [55] detected DNA damage in paint workers with low toluene exposure using the COMET assay, but MN frequency remained unchanged. Cavallo et al. [56], used the Fpg-COMET assay to confirm both direct and oxidative DNA damage in shipyard painters, along with elevated urinary 8-oxoGua and decreased 8-oxodGuo and 8-oxoGuo levels. Among roller painters with relatively lower VOC exposure, increased cytogenotoxicity was observed only with the BMCyt test, which was attributed to task-specific exposure levels and use of personal protective equipment (PPE). Finally, Martino Roth et al. [35], reported genotoxic risk using both MN and COMET assays in ten automotive painters, while Cárdenas-Bustamante et al. [30], found no significant differences in genotoxic endpoints despite solvent exposure.

A recent study by Çetintepe et al. [28], investigated the effects of multiple chemical exposures on genetic damage and oxidative stress among automotive paint workers in Ankara. The alkaline COMET assay was applied to peripheral blood lymphocyte samples from 110 exposed workers and 60 unexposed controls, revealing significantly elevated DNA damage in the worker group. In addition, key oxidative stress biomarkers including glutathione peroxidase (GPx), glutathione reductase (GR), and glutathione (GSH) were significantly decreased in exposed individuals. Conversely, levels of malondialdehyde (MDA) and 8-hydroxy-2-deoxyguanosine (8-OHdG), both indicative of lipid peroxidation and oxidative DNA damage, were significantly elevated. The study also demonstrated increased levels of pro-inflammatory cytokines such as TNF-α, IL-1β, IL-17, IL-23, and Clara cell protein (CC16), suggesting activation of inflammatory pathways. These findings underscore the multidimensional toxicity associated with chemical exposures in the paint industry, implicating not only genomic instability but also disruptions to oxidative balance and immune regulation. The results are consistent with our study, which similarly identified increased genotoxicity using both COMET and MN assays in paint workers. Together, these findings emphasize the value of integrated biomonitoring approaches that combine genotoxicity, oxidative stress, and inflammatory biomarkers for a more comprehensive risk assessment. However, it is important to acknowledge certain limitations in the study by Çetintepe et al. [28]. Quantitative assessment of individual chemical exposure levels was not conducted, which limited the ability to establish dose–response relationships. Furthermore, reliance on self-reported occupational histories introduces the potential for recall bias or misclassification. Despite these limitations, the combined application of COMET and MN assays provides a robust and complementary assessment of the biological effects of occupational exposures, as previously noted by Leandro et al. [37].

## 5. Conclusions

This study demonstrated that paint workers exhibited significantly increased DNA damage in both peripheral blood lymphocytes and whole blood, as measured by the alkaline COMET assay. Additionally, elevated frequencies of MN, BN, chromatin condensation, karyorrhexis, and nuclear budding were observed in exfoliated buccal epithelial cells. These findings indicate that occupational exposure to VOCs, organic solvents, and heavy metals in the paint industry results in substantial genotoxic effects.

The observed genotoxic alterations suggest that individuals employed in the paint sector are at increased risk of genetic damage, underscoring the urgent need for regular biological monitoring and the implementation of effective occupational health and safety measures. Notably, the complex chemical mixtures encountered in paint-related tasks may exert synergistic toxic effects, where the combined action of two or more compounds produces greater harm than each individually.

Given these risks, it is imperative to enforce strict occupational health policies, ensure access to PPE, and provide comprehensive worker training on chemical safety. Periodic biomonitoring using validated assays—such as the COMET and MN tests—should be standard practice in high-risk occupational settings.

Furthermore, the findings of this study serve as a critical warning for other occupational groups exposed to similar chemical environments. Future research should aim to elucidate the molecular mechanisms of genotoxicity, explore potential epigenetic modifications, and assess long-term health outcomes through longitudinal studies with larger and multicenter participation to allow for robust multivariate modeling. These efforts will contribute to a deeper understanding of the cumulative and chronic health effects of complex chemical exposures in the workplace.

## Figures and Tables

**Table 1:** Demographic characteristics of the study population.

Factors	Controls (n = 80)	Workers (n = 80)	*p*
Age (years) ^a^	37.83 ± 8.16	39 ± 7.17	non-significant *
19–34	19 (23.75% **)	17 (21.25% **)	non-significant ***
≥35	61 (76.25% **)	63 (78.75% **)	
Smokers	28 (35% ***)	45 (56.25% ***)	0.006 ***
Non-smokers	52 (65%*)	35 (43.75% ***)	
Alcohol consumption			Non applicable
Yes	0 (0% ***)	0 (0% ***)	
No	80 (100% ***)	80 (100% ***)	
Duration of Exposure (years)			Non applicable
1–17	0 (0% *)	63 (78.75%)	
18–35	0 (0% *)	17 (21.25%)	

^a^ Expressed as Mean ± SD, SD: standard deviation. * Student T test was used. ** Column proportion. *** Pearson Chi Square test was used.

**Table 2:** Assessment of lymphocyte and whole blood DNA damage in worker and control populations.

Factors	Tail Intensity in Lymphocytes (%)		*p* **	Tail Intensity in Whole Blood (%)		*p* **
	Controls (n = 80)	Workers (n = 80)		Controls (n = 80) ^a^	Workers (n = 80) ^a^	
All groups	2.69 ± 1.05	5.00 ± 0.50 *	<0.05	5.48 ± 2.41	7.65 ± 3.47	<0.05
Age						
19–34	2.14 ± 0.97	4.58 ± 4.22	non-significant	4.82 ± 2.41	8.75 ± 3.39	non-significant
≥35	2.89 ± 1.01	6.16 ± 1.93	non-significant	5.74 ± 2.04	7.36 ± 3.46	non-significant
Smokers	2.53 ± 1.18	5.91 ± 5.60	non-significant	5.01 ± 2.50	8.50 ± 3.70	non-significant
Non-smokers	2.78 ± 0.97	4.26 ± 4.52	non-significant	5.76 ± 2.34	6.98 ± 3.16	non-significant
Duration of Exposure (years)	**-**		n/a	**-**		n/a
1–17		5.19 ± 0.29			7.50 ± 3.58	
18–35		4.35 ± 4.26			8.24 ± 3.07	

^a^ Expressed as mean ± SD; SD: standard deviation. * *p* < 0.05 compared to controls. ** Student T test was used.

**Table 3:** Buccal MN frequencies among workers and control group ^a^.

Factors	Buccal MN Frequencies (‰)		*p* *
	Controls (n = 80) ^a^	Workers (n = 80) ^a^	
All groups	2.36 ± 1.93	3.23 ± 2.71	<0.05
Age			
19–34	2.36 ± 2.17	2.24 ± 2.14	non-significant
≥35	2.37 ± 1.85	3.52 ± 4.01	non-significant
Smokers	2.64 ± 1.98	3.30 ± 4.08	non-significant
Non-smokers	2.23 ± 1.92	3.16 ± 3.20	non-significant
Duration of Exposure (years)			
1–17	**-**	2.93 ± 1.41	n/a
18–35	**-**	4.38 ± 4.63	n/a

^a^ Expressed as mean ± SD; SD: standard deviation. * Student T test was used.

**Table 4:** Cytogenetic abnormalities and biomarker levels in buccal cells and biological samples of paint workers and control subjects.

		Controls (n = 80) ^a^	Workers (n = 80) ^a^	*p* *
Cell frequencies (‰)	BN	4.80 ± 3.25	29.23 ± 10.32	*p* < 0.01
	CC	5.40 ± 3.63	20.55 ± 18.18	*p* < 0.01
	KHC	2.33 ± 3.65	4.76 ± 1.84	*p* < 0.05
	KYL	11.50 ± 11.10	49.99 ± 5.99	*p* < 0.01
	PYC	4.38 ± 3.51	21.57 ± 4.06	*p* < 0.05
	NBUD	0.48 ± 0.68	0.71 ± 0.42	*p* < 0.05
Biomarker Analysis	TCA	2.01 ± 1.30	8.62 ± 6.13	*p* < 0.05
	H. ACID	212.7 ± 78.38	1396.66 ± 791.71	*p* < 0.01
	PHENOL	1.40 ± 0.31	10.94 ± 20.18	*p* < 0.05
	Pb	1.16 ± 0.45	3.40 ± 3.72	*p* < 0.05

^a^ Expressed as mean ± SD; BN: binucleated; CC: condensed chromatin; KHC: karyorrhectic; KYL: karyolytic; PYC: pyknotic; NBUD: nuclear bud; TCA: trichloroacetic acid; H.ACID: hippuric acid; Pb: blood lead levels; SD: standard deviation.* Student T Test was used.

## Data Availability

The data presented in this study are available on request from the corresponding author. The data are not publicly available due to ethical restrictions.
